# Novel Alkylimidazolium Ionic Liquids as an Antibacterial Alternative to Pathogens of the Skin and Soft Tissue Infections

**DOI:** 10.3390/molecules23092354

**Published:** 2018-09-14

**Authors:** Oscar Forero Doria, Ricardo Castro, Margarita Gutierrez, Diego Gonzalez Valenzuela, Leonardo Santos, David Ramirez, Luis Guzman

**Affiliations:** 1 Instituto de Química de Recursos Naturales, Universidad de Talca, P.O. Box 747, Talca 3460000, Chile; oforero@utalca.cl (O.F.D.); mgutierrez@utalca.cl (M.G.); lssantos@utalca.cl (L.S.); 2 Multidisciplinary Agroindustry Research Laboratory, Universidad Autónoma de Chile, Talca 3460000, Chile; ricastro@utalca.cl; 3 Carrera de Ingeniería en Construcción e Instituto de Ciencias Químicas Aplicadas, Universidad Autónoma de Chile, Talca 3460000, Chile; 4 Departamento de Salud Municipal de Hualañe, Maule 3530000, Chile; dgonzalez@ssmaule.cl; 5 Instituto de Ciencias Biomédicas, Universidad Autónoma de Chile, Talca 3460000, Chile; damach.david@gmail.com; 6 Departamento de Bioquímica Clínica e Inmunohematología, Facultad de Ciencias de la Salud, Universidad de Talca, P.O. Box 747, Talca 3460000, Chile

**Keywords:** ionic liquids, antibacterial, skin and soft tissue infections

## Abstract

Keeping in mind the concept of green chemistry, this research aims to synthesize and characterize new ionic liquids (ILs) derived from *N*-cinnamyl imidazole with different sizes of alkyl chains (1, 6, 8, and 10 carbon atoms), and evaluate their antibacterial activity against Skin and soft tissue infections (SSTIs) causative bacteria. The antibacterial screening was carried out by agar well diffusion and the Minimum Inhibitory Concentration (MIC) and Half Maximum Inhibitory Concentration (IC_50_) of the different ILs were determined by microdilution in broth, also Molecular dynamics simulations were performed to study the interaction mechanism between ILs and membranes. The MIC value in Gram-positive bacteria showed that as the hydrocarbon chain increases, the MIC value decreases with a dose-dependent effect. Furthermore, Gram-negative bacteria showed high MIC values, which were also evidenced in the antibacterial screening. The molecular dynamics showed an incorporation of the ILs with the longer chain (10 C), corresponding to a passive diffusion towards the membrane surface, for its part, the ILs with the shorter chain due to its lack of hydrophobicity was not incorporated into the bilayer. Finally, the new ILs synthesized could be an alternative for the treatment of Gram-positive bacteria causative of SSTIs.

## 1. Introduction

Skin and soft tissue infections (SSTIs) are a worldwide public health problem considered as an important cause of absenteeism; having thus unfavorable consequences for companies, workers, and families [1]. SSTIs must be dealt with during illness because carriers often require hospitalization, leading to a non-negligible morbidity [2].

As reported by health services, the main SSTIs etiological agents are *Escherichia coli*, enterobacterias *Pseudomonas aeruginosa* (macerated ulcers), *Staphylococcus aureus* (methicillin-resistant *S. aureus* in previously treated ulcers), *Streptococcus pyogenes*, and *Staphylococcus* spp. coagulase negative (*Staphylococcus epidermidis* in immunosuppressed patients). It has been stated that one out of three SSTIs is caused by a Gram-positive cocci, while the rest are mainly caused by *E. coli* and *P. aeruginosa* [3].

The World Health Organization of the United Nations (WHO-UN) has reported an increase in bacterial resistance in SSTIs. Nowadays, this organization has alerted and kept surveillance over these resistances to control them. The most substantial reports of this phenomenon are regarding Gram-negative pathogens such as *E. coli* and *Klebsiella pneumoniae* [4,5].

Hence, it is critical to generate new antibacterial alternatives against SSTIs agents. Among some of the most innovative compounds of our times are Ionic Liquids (ILs). These compounds are salts based on complex ions, whose phase is liquid at room temperature. Their physicochemical properties deliver low vapor pressure characteristics, along with the generation of multiple solvation layers; allowing thus the interaction between organic and inorganic molecules [6]. These compounds also exhibit excellent thermochemical stability and electrochemical conductivity [7].

Furthermore, properties such as viscosity, hydrophobicity, and density can be managed by modifying their nuclei and their solubility can be altered by combining hydrophobic chains or hydrophilic functional groups [8]. The solubility can be changed dramatically by the choice of anions, such as halide (Cl^−^, Br^−^, I^−^), nitrate (NO_3_^−^), acetate (CH_3_CO_2_^−^), tetrafluoroborate (BF_4_^−^), triflate (CF_3_SO_3_^−^) bis (trifluoromethylsulfonyl) imide (CF_3_SO_2_)_2_N^−^ and hexafluorophosphate (PF_6_^−^), among others. Seddon et al., 2000 [9], reported some general guidelines about the effect of the anion in the miscibility of the ILs and water, showing that imidazolium salts with halide, acetate, nitrate and trifluoroacetate anions are totally miscible in water, while imidazolium salts containing PF_6_^−^ and (CF_3_SO_2_)_2_N^−^ anions are immiscible in water. On the other hand, imidazolium salts with BF_4_^−^ and CF_3_SO_3_^−^ anions can be totally miscible or immiscible depending on the substituents on the cation.

Besides the great structural diversity that can be achieved, nowadays it is also necessary to consider the toxicity, biodegradation, and bioaccumulation of these molecules to determine the potential environmental impact. Preliminary toxicity data are advisable and biodegradation studies can determine the possible toxicity of the IL and/or their metabolite(s); these results are very important since an IL that does not pass the biodegradation test has the potential to bioaccumulate. To ensure that an IL and its metabolites will not persist in nature, its recommendable to afford biodegradation, mineralization, and bioaccumulation studies [10,11].

In the 90’s, ILs were produced using techniques such as metathesis, acid-base neutralization or by a direct combination. Nowadays, nature and environmental conservation are a matter of concern for the chemical sciences. Therefore, the use of microwaves appears to be an important tool determined to reduce the environmental impact and to enhance people’s health; leading to the international concept of “green chemistry” [12,13]. Synthetic chemistry seeks to standardize conditions and replicate methods over time; therefore the use of microwaves (when controlled) as a method for delivering stable temperatures to the system, reducing reaction time, and eliminating or reducing highly toxic organic solvents used by operators, which would also end up endangering the environment [14,15], are important methods to implement.

Bottom line, the obtention of novel antibacterial alternatives for agents causing SSTIs is crucial. Keeping in mind the concept of green chemistry, this research project aims to synthesize, characterize, and evaluate new water-soluble ILs as antibacterial agents against SSTIs causative bacteria.

## 2. Results

### 2.1. Chemistry

**Scheme 1:**
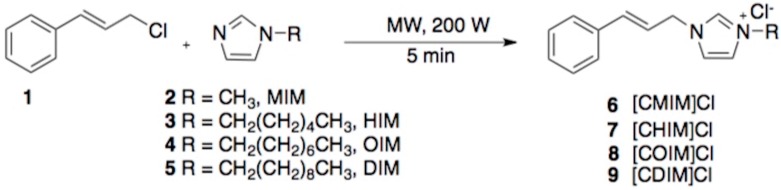
Synthesis of different ILs derived from cinnamyl with different chain lengths.

Conventional methods of ILs construction by heating generally involve several hours of reaction, high-energy consumption, and require a large excess of organic solvents. Motivated by safe and eco-friendly protocols to obtain the ionic liquids, here we report on the construction of different *N*-cinnamylimidazolium salts employing microwave (MW) radiation under a solvent-free condition as a Green chemistry approach. In this sense, the construction of *N*-cinnamylmidazolium salts with different alkyl size chains (1, 6, 8, and 10 carbon atoms) was synthesized by reacting cinnamyl chloride with different alkylimidazoles (MIM, HIM, OIM, and DIM) (Scheme 1). Resulting ILs were then cooled to room temperature and their solubility was tested in EtOAc, MeOH, and H_2_O. *N*-cinnamylimidazolium salts were soluble in MeOH and H_2_O; thereupon, they were rinsed with EtOAc to remove the starting reagents, and finally concentrated under high vacuum to afford [CMIM]Cl (**6**), [CHIM]Cl (**7**), [COIM]Cl (**8**), and [CDIM]Cl (**9**).

### 2.2. Fourier-Transform Infrared Spectroscopy (FT-IR)

**Figure 1:**
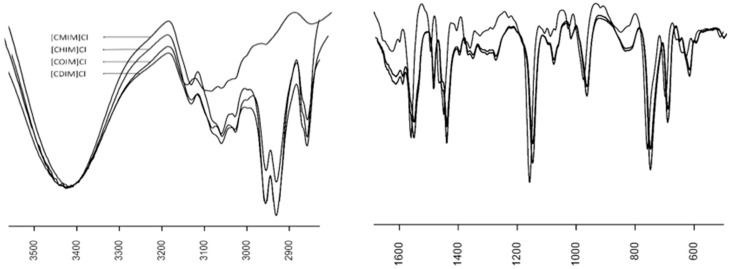
IR spectra of the different *N*-cinnamylimidazolium ILs.

IR spectra (Figure 1) for all synthesized *N*-cinnamylimidazolium salts showed a band at 3300–3600 cm^−1^ assigned to the bending and stretching modes of absorbed water, stretching absorption bands at 3100–3060 cm^−1^, 2960–2930 cm^−1^ [16], 1630–1570 cm^−1^ [17] and 1289–774 cm^−1^ attributed to (C-H) Aromatic, (C-H) Aliphatic, (C=N), and (C=C) Aromatic (ring-breathing modes) [18], respectively. It is worthwhile to highlight that the absorption band at 2960–2930 cm^−1^ (characteristic of the methylene group (-CH_2_-)) is more intense as the alkyl chain grows. Thus, the signal intensity in increasing order for the different *N*-cinnamylimidazolium ILs was [CMIM]Cl < [CHIM]Cl < [COIM]Cl < [CDIM]Cl.

### 2.3. Thermal Stability

**Figure 2:**
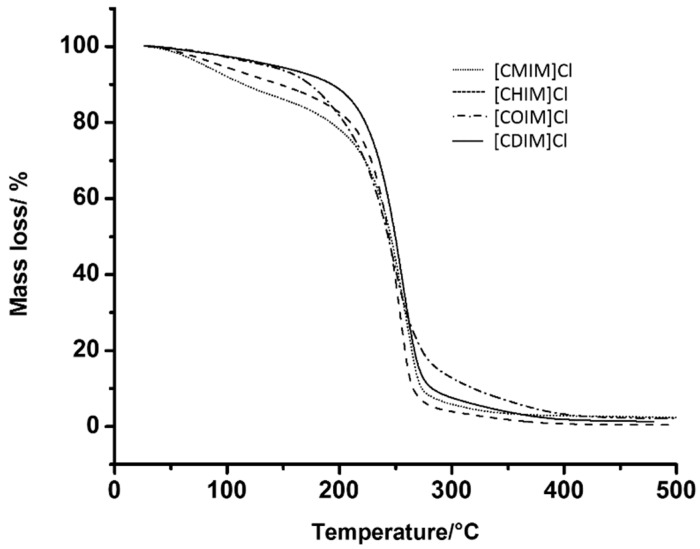
TG curve of different *N*-cinnamylimidazolium ILs.

To study the thermal stability of the different ILs presented here, thermal analyses were undertaken in an interval of 20–700 °C. The mass loss curve (TG) is presented in Figure 2. The observed thermal behavior is very similar in all ILs synthesized and their onset temperature (T_onset_/°C: Degradation start temperature) was very low (Table 1). Moreover, an increase in thermal stability was observed as the carbon chain length grew, being [CDIM]Cl the most stable at T_onset_/°C of 106 °C, and [CMIM]Cl the least stable.

### 2.4. Antibacterial Screening

**Figure 3:**
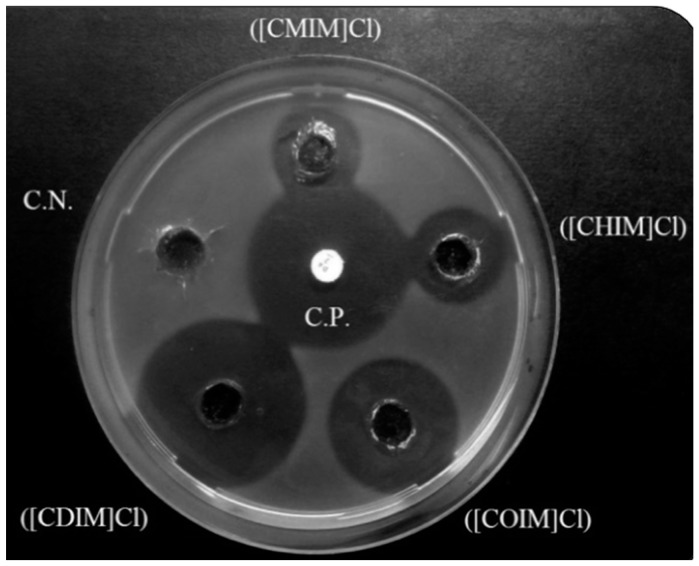
*S. aureus* growth inhibition halos in the presence of ILs with different chain lengths. C.P. positive control (mupirocin 5 μg), C.N. negative control.

All ILs were tested for their antibacterial activity using the agar well diffusion qualitative method. As an example, Figure 3 shows the inhibition halos results of *S. aureus* bacterial growth. The entire outcomes are shown in Table 2. As the chain length increases, the bacterial sensitivity also increases; this phenomenon is clearly seen in Gram-positive cocaceae, where all were inhibited in at least three concentrations of *N*-cinnamylalkylimidazolium ILs. Likewise, the same phenomenon but to a lesser extent was observed in *E. coli* and *A. baumannii*.

### 2.5. Determination of Minimum Inhibitory Concentration (MIC) and Mean Inhibition of Bacterial Viability (IC_50_)

Minimal inhibitory concentrations (MIC) were determined by colorimetric assays and reported as the last dilution where the pathogen generates metabolic activity. MIC value in Gram-positive cocaceas (Table 3) range from 31.25 μM to 250 μM, showing that, as the hydrocarbon chain increases, the MIC value decreases with a dose-dependent effect. Furthermore, Gram-negative bacteria showed high MIC values (Table 3), which were also evidenced in the previous antibacterial screening. Only *P. aeruginosa* presents resistance to ILs out of all Gram-negative bacteria studied, whereas *E. coli* had MIC values of 250 μM and *A. baumannii* of 1000 μM. Besides, an effect based on the chain length and the ILs concentration was observed in Gram-positive bacteria.

IC_50_’s (Table 3) data were obtained from the log graphs of the ILs concentration versus the inhibition percentage measured at 12 h. Obtained graphs correspond to a statistical normalization of a dose-response effect. The salts inhibitory activity derived from *N*-Cinnamyl imidazole against bacteria *S. aureus*, *S. pyogenes*, *S. epidermidis*, *E. coli,* and *A. baumannii* was determined; however, it was not possible to calculate the IC_50_ for *P. aeruginosa* given the resistance previously indicated.

For each case, an inhibition of the bacterial viability in a dose-dependent manner was observed, since the increasing of ILs concentration also increases the percentage of bacterial inhibition. It was evidenced that the Gram-positive bacteria were more sensitive to these ILs since the graph’s point of inflection was at a lower concentration than the Gram-negative species.

### 2.6. Molecular Dynamics Simulations (MDs) and ADME Properties

**Figure 4:**
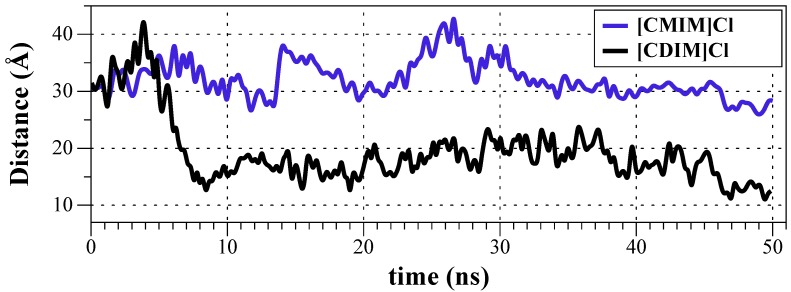
Distances between ILs [CDIM]Cl (black) and [CMIM]Cl (blue) and membrane center of masses (COMs) as a function of simulation time.

**Figure 5:**
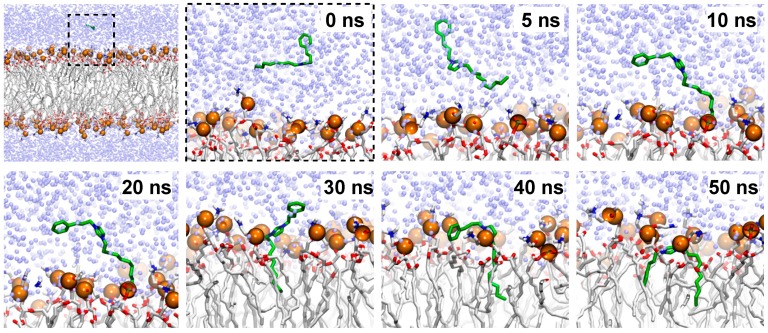
Incorporation of [CDIM]Cl into the membrane along with the 50 ns MDs.

MDs for ILs in a DOPC bilayer were performed to study their incorporation into the lipid phase. According to the antibacterial screening and biological activity determinations, ILs with longer aliphatic chains are expected to be incorporated into the lipid bilayer. Ligands were immersed in bulk water by setting at 30 Å the distance between the ligand and the lipid center of mass. [CDIM]Cl was incorporated into the bilayer and remained within the membrane for the rest of the simulation time after 8 ns; this phenomenon corresponds to the passive diffusion of [CDIM]Cl towards the membrane surface. [CMIM]Cl interacted with the membrane polar head groups along with the 50 ns of MDs, but due to its lack of hydrophobicity the compound was not incorporated into the bilayer (Figure 4). Following the [CDIM]Cl incorporation into the membrane, the imidazole polar ring (Figure 5) interacted (at membrane’s glycerol level) with the aliphatic moieties interrelating with the lipid alkyl chains (Figure 5 and Supplemental Movie S1).

ADME properties were predicted (Table 4 and Table 5), and results suggested that there are no significant violations of Lipinski’s rule since all calculated physicochemical descriptors and pharmacokinetics properties are within the expected thresholds (molecular weight (g/mol) = 200–326; Log P = 1.859–4.523; HB acceptors = 4 and HB donors = 0). Additionally, PSA and water solubility (log S) parameters, which are important in the membrane penetration, were analyzed and the results were within the acceptable range established for human use [19].

## 3. Discussion

Regarding ILs thermal characterization, it is important to highlight that the counter ion plays a very important role in the thermal stability of an ionic liquid. Erdmenger et al. [20] demonstrated that imidazolium chloride ILs present a low decomposition with T_onset_/°C values in the range of 48–80 °C. In contrast, the change of the counter ion for others of a different nature as tetrafluoroborate (BF_4_^−^) increases the thermal stability. These results are like the values (T_onset_: 61–106 °C) reported here for *N*-cinnamylimidazolium ILs, which could be due to the presence of unsaturated groups in its structure. Similar observations were reported by Anderson et al. [21], their results show that Imidazolium and Benzylimidazolium geminal dicationic ILs, incorporating unsaturated side groups showed lower thermal stability than their fully saturated analogues; also, Al-Mohammed et al. [22] observed these variations in the decomposition temperatures. Additionally, the thermal analysis of the synthesized ILs showed that when more asymmetrical is its structure, the decomposition temperatures are much lower and it is more stable when it presents a side chain of 10 carbon atoms, which is probably explained by the force of the interaction between the cation and anion, being this interaction is much weaker when its structure is more asymmetrical, hence its decomposition temperatures are much lower [23].

Conversely, Tröger-Müller et al. [24] determined the correlation between different counter ions and the thermal stability of an ionic liquid. Thus, the decreasing order of ILs thermal behavior based on the counter ion was tetrafluoroborate > hexafluorophosphate > chloride > organic acids. Moreover, alkyl chains length play a fundamental role in the thermal stability of an ionic liquid, observing that by increasing the chain size, its maximum temperature of degradation also increases. In this sense, for the *N*-cinnamylimidazolium ILs reported here, the thermal stability increasing order was as follows [CMIM]Cl < [CHIM]Cl < [COIM]Cl < [CDIM]Cl. This behavior can be observed at T_10_/°C and T_50_/°C mass loss temperature of the different *N*-cinnamylimidazolium ILs, realizing that the temperature increases in the range of 118–193 and 245–257 °C, respectively.

Antibacterial screening against the Gram-positive cocaceae is similar to other salts derived from imidazole. Studies were carried out using ILs with structural similarity than those from Anvari et al., who described an antibacterial activity with inhibition halos over 20 mm for *S. aureus* and *E. coli* [25]. Busetti et al. reported antibacterial activities of 1-alkyl-quinoline ([Cnquin]Br), a structure-like IL similar to derivatives of *N*-acetylimidazole with chain lengths of 8, 10, 12, 14, 16 and 18 carbons, indicates that the chain length increasing would also increase the antimicrobial activity [26]. Researchers reported a MIC of 121.2 μM for *S. aureus* with a chain length of 10 carbon atoms; for the same bacteria, the synthesized IL [CDIM]Cl showed a MIC of 31.25 μM. Meanwhile, Cornellas et al. reported activities of 1-alkylpyridine bromide (CnPyrBr) against *S. epidermidis*, with a MIC = 940 μM [27]. In this study, we are reporting an antibacterial activity of MIC = 250 μM in the derivative [CMIM]Cl (shorter chain) against *S. epidermidis*, proving that the chain length is not the only feature important for antibacterial activity. Regarding this statement, Luczak et al., have estimated that chains of 10 carbon atoms or more would induce a lower tolerance of human cells due to the surface tension generated by the ILs, which would affect its viability by causing a membrane shock [28].

Ferraz et al., reported ILs antibacterial activity against *S. epidermidis* and *S. aureus*. The study revealed that the MICs were able to decrease when the ILs were combined with antibiotics ([C16Pyr] [Cl] with a MIC value of 0.5 mM for *S. aureus* and [C16Pyr] [Amp] (ampicillin-coupled LI) with a MIC value of 0.005 mM [29]. These results are coherent with several investigations where ILs were used as carriers for proteins or another molecule in plasma [30,31]. ILs physical and chemical characteristics that are assessed in this work could help in the solubility of improving antibiotics with a hydrophobic nature since hydrocarbon chains provide the lipophilia property.

Results obtained for Gram-positive bacteria are promising since the bacterial viability IC_50_ was about 25–140 μM; being lower than that reported by other authors [25,29,32]. Conversely, results for Gram-negative bacteria were less promising with a higher MIC and IC_50_ values. However, these results are consistent with the ones reported about the natural resistance of Gram-negative bacteria to ILs [25,29,32,33]. Gram-negative bacteria resistance is expressed by multiple factors affecting not only the ILs but also the antibiotics used for the treatment of infections [34]. These bacteria can express molecular expulsion pumps and modify their target sites to block drugs; besides, they can also express hydrolytic enzymes and membrane proteins that would give resistance to osmotic shock [35,36]. Ferraz et al., coupled ILs to antibiotics for Gram-positive bacteria, revealing a substantial antibiotic improvement in the antibacterial activity against *S. aureus* with a MIC value of 100-fold lower (compared to the unconjugated antibiotic). These results were not extrapolated to Gram-negative bacteria [29].

Despite the eco-friendly synthesis, there are still are other concerns to be considered on further studies regarding the possible ecological effects of these molecules. ILs have the potential to affect ecosystems through different mechanisms, among them the mortality of individual organisms, altered biogeochemical processes, or bioaccumulation in higher trophic levels. Studies of the acute and chronic toxicities of imidazolium-based ILs to the water flea *Daphnia magna* have showed that the most toxic IL (acute toxicity) was the 1-butyl-3-methylimidazolium bromide ([bmim]Br) with a LC_50_ of 8.03 mg L^−1^, because the lethal concentrations are much lower for ILs with imidazolium as the cation than for salts with Na as the cation, for its part, the 1-butyl-3-methylimidazolium chloride ([bmim]Cl) showed an intermediate toxicity with a LC_50_ of 14.80 mg L^−1^, suggesting that the toxicity was related to the imidazolium cation and not to the anions tested [37]. Mohedano et al., using respiration inhibition assays with activated sludge, found in most of the cases a well-fitting linear correlation between the toxicity (logEC_50_) and the length of the alkyl chain (C_4_–C_10_) of imidazolium ILs; the toxicity increases significantly with the chain length, which can be related to the loss of IL polarity or their lipophilic character. The bis (trifluoromethanesulfonyl) imide (NTf_2_^−^) anion was more toxic than tetrafluoroborate (BF_4_^-^) or chloride (Cl), but its relative impact on toxicity is reduced for ILs with long alkyl side chain (>6 *C* atoms) [38]. At the same time, Wang et al., using different imidazole nitrate ILs at concentrations of 5, 10, 20, and 40 mg kg^−1^ of ILs, showed that all of them caused oxidative stress and oxidative damage in earthworms (*Eisenia fetida*), evidenced in the level of biomarkers as reactive oxygen species (ROS) and malondialdehyde (MDA) content. Nevertheless, the toxicity of these five ILs did not correlate with the chain length, showing an order of toxicity of [C_10_mim]NO_3_ < [C_12_mim]NO_3_ < [C_4_mim]NO_3_ < [C_6_mim]NO_3_ < [C_8_mim]NO_3_ [39]. In our case, the functionalization of the ILs with a cinnamyl group could lead to an improvement in the MIC and IC values as discussed previously, also the presence of this cinnamic acid derivate could prevent some of the deleterious effects (e.g., oxidative stress) observed in other imidazolium ILs, which only have a methyl group.

Molecular dynamics simulation results of the ILs insertion in a DOPC double layer indicate that the substituted imidazole moiety interacts with the glycerol moieties at the membrane surface. While the [CDIM]Cl aliphatic chain extends to the interior of the membrane, the [CMIM]Cl lacking hydrophobic makes it more difficult to interact with the membrane for a subsequent diffusion to the intracellular space. These results confirm the hypothesis where the greater the ILs apolar chain is, the better the interaction with the membrane will be, thus giving a better antimicrobial activity. All physical and pharmaceutical properties calculated for the ILs studied here are within the acceptable range defined for human use, indicating thereby, their potential use as new antibacterial alternatives against SSTIs agents. ILs computational analysis confirms that the lipophilicity is crucial for drug-membrane interaction [40]. This property is one of the most important drug-like properties with a great impact in physicochemical and pharmacokinetic processes.

## 4. Material and Methods

### 4.1. General Information

Reagents for synthesis were obtained from Sigma-Aldrich (St. Louis, MO, USA). ^1^H and ^13^C-NMR spectra (400 MHz for proton and 100 MHz for carbon) were recorded on an AM-400 spectrometer (Bruker, Rheinstetten, Germany); IR spectra (KBr pellets, 500–4000 cm^−1^) were recorded on a NEXUS 670 FT-IR spectrophotometer (Thermo Nicolet, Madison, WI, USA). Mass spectrometry was conducted in a High resolution mass spectrometer Exactive™ Plus Orbitrap (ThermoFisher Scientific, Bremen, Germany), Scan parameters: Resolution: 140,000, AGC target: 3e6, Max. inject time: 200, HESI source: Sheath gas flow: 10, Aux gas flow rate: 3, Sweep gas flow rate: 0, Capillary temp.: 250 °C, S-lens RF level: 0, Heater temp: 50 °C.

### 4.2. Chemistry

#### 4.2.1. General Procedure for Alkylimidazoles Synthesis

Briefly, a mixture of imidazole (100 mmol), alkyl bromide (100 mmol) and K_2_CO_3_ (200 mmol) in acetone (200 mL) was refluxed overnight. Upon filtration and solvent removal, the remaining residue was subjected to flash chromatography with ethyl acetate to produce >90% yield.

#### 4.2.2. General Procedure for the Construction of Different *N*-Cinnamylimidazolium Ionic Liquids

Briefly, a mixture of alkylimidazoles such as 1-methyl-1*H*-imidazole (MIM), 1-hexyl-1*H*-imidazole (HIM), 1-octyl-1*H*-imidazole (OIM), and 1-decyl-1*H*-imidazole (DIM) (1 mmol), as well as cinnamyl chloride (1 mmol) solvent-free were microwaved to 200 MW at 80 °C for 5 min (optimum reaction condition). Reaction completion was marked by separation of dense IL. Products such as 1-methyl-3-cinnamylimidazolium chloride [CMIM]Cl, 1-hexyl-3-cinnamylimidazolium chloride [CHIM]Cl, 1-octyl-3-cinnamylimidazolium chloride [COIM]Cl, and 1-decyl-3-cinnamylimidazolium chloride [CDIM]Cl were isolated by decanting toluene to remove any unreacted starting materials and solvents. Subsequently, ILs were rinsed with diethyl ether (4 × 10 mL) separating afterward this latter layer by decantation. In each case, ILs were finally dried under reduced pressure to get rid of the volatile organic compounds.

*1-Methyl-1 H-imidazole (MIM)* (**2**). 90% yield. ^1^H NMR (400 MHz, CDCl_3_) δ 7.60 (s, 1H), 7.21 (s, 1H), 7.06 (s, 1H), 3.84 (s, 3H); ^13^C NMR (100 MHz, CDCl_3_) δ 137.5, 128.9, 120.0, 33.0.

*1-Hexyl-1 H-imidazole (HIM)* (**3**). 85% yield. ^1^H NMR (400 MHz, CDCl_3_) δ 7.67 (s, 1H), 7.26 (s, 1H), 7.12 (s, 1H), 4.14 (t, *J* = 7.1 Hz, 2H), 2.09–1.87 (m, 2H), 1.50 (d, *J* = 10.0 Hz, 7H), 1.10 (s, 3H); ^13^C NMR (100 MHz, CDCl_3_) δ 136.6, 128.4, 118.4, 46.6, 30.8, 30.6, 25.8, 22.0, 13.5.

*1-Octyl-1 H-imidazole (OIM)* (**4**). 80% yield. ^1^H NMR (400 MHz, CDCl_3_) δ 7.67 (s, 1H), 7.26 (s, 1H), 7.12 (s, 1H), 4.13 (t, *J* = 7.1 Hz, 2H), 2.06–1.86 (m, 2H), 1.50 (d, *J* = 9.6 Hz, 10H), 1.10 (s, 3H); ^13^C NMR (100 MHz, CDCl_3_) δ 136.7, 128.9, 118.5, 46.7, 31.4, 30.8, 28.8, 28.7, 26.2, 22.3, 13.7.

*1-Decyl-1 H-imidazole (DIM)* (**5**). 70% yield. ^1^H NMR (400 MHz, CDCl_3_) δ 7.67 (s, 1H), 7.26 (s, 1H), 7.12 (s, 1H), 4.13 (t, *J* = 7.1 Hz, 2H), 2.09–1.86 (m, 2H), 1.49 (d, *J* = 11.9 Hz, 14H), 1.09 (t, *J* = 6.4 Hz, 3H); ^13^C NMR (100 MHz, CDCl_3_) δ 137.0, 129.3, 118.8, 47.0, 31.8, 31.1, 29.5, 29.4, 29.2, 29.1, 26.5, 22.6, 14.1.

*1-Methyl-3-cinnamylimidazolium chloride* (**6**). 90% yield. ^1^H NMR (400 MHz, MeOD) δ 9.03, 8.62 (s, each 0.5H), 7.63 (m, 2H), 7.50–7.20 (m, 5H), 6.86 (d, *J* = 15.8 Hz, 1H), 6.53–6.38 (m, 1H), 5.09–4.93 (m, 2H), 3.94 (s, 3H); ^13^C NMR (100 MHz, MeOD) δ 138.1, 138.0, 137.0, 129.8, 129.7, 128.0, 127.85, 122.2, 52.6, 52.5, 51.5, 36.6, 35.7. HRMS (HESI/Orbitrap) *m*/*z* calculated for C_13_H_15_ClN_2_ [CMIM]^+^: 199.1230, found 199.1230.

*1-Hexyl-3-cinnamylimidazolium chloride* (**7**). 95% yield. ^1^H NMR (400 MHz, MeOD) δ 9.13, 8.95 (s, each 0.5H), 7.68 (s, 2H), 7.55–7.03 (m, 5H), 6.85 (dt, *J* = 15.3, 7.6 Hz, 1H), 6.45 (dd, *J* = 15.2, 6.8 Hz, 1H), 5.02 (d, *J* = 6.3 Hz, 2H), 4.37–3.95 (m, 2H), 1.89 (s, 2H), 1.34 (s, 6H), 0.90 (s, 3H); ^13^C NMR (100 MHz, MeOD) δ 138.0, 137.2, 136.9, 129.8, 128.0, 123.2, 122.2, 121.4, 52.6, 51.0, 50.9, 50.5, 32.2, 31.2, 31.1, 26.9, 23.4, 14.3. HRMS (HESI/Orbitrap) *m*/*z* calculated for C_18_H_25_ClN_2_ [CHIM]^+^: 269.2018, found 269.2012.

*1-Octyl-3-cinnamylimidazolium chloride* (**8**). 85% yield. ^1^H NMR (400 MHz, MeOD) δ 9.17, 8.42 (s, each 0.5 H), 7.73 (s, 2H), 7.55–7.29 (m, 5H), 6.89 (d, *J* = 15.8 Hz, 1H), 6.62–6.36 (m, 1H), 5.06 (d, *J* = 6.7 Hz, 2H), 4.27 (t, *J* = 7.2 Hz, 2H), 1.93 (s, 2H), 1.38 (s, 7H), 0.93 (s, 3H); ^13^C NMR (100 MHz, MeOD) δ 138.0, 137.1, 136.8, 129.8, 129.6, 127.9, 124.5, 123.8, 123.6, 122.1, 122.0, 52.4, 50.8, 32.1, 31.4, 31.0, 26.9, 26.8, 23.4, 14.2. HRMS (HESI/Orbitrap) *m*/*z* calculated for C_20_H_29_ClN_2_ [COIM]^+^: 297.2325, found 297.2321.

*1-Decyl-3-cinnamylimidazolium chloride* (**9**). 90% yield. ^1^H NMR (400 MHz, MeOD) δ 9.14 (s, 1H), 7.71 (s, 2H), 7.53–7.26 (m, 5H), 6.87 (d, *J* = 15.8 Hz, 1H), 6.61–6.29 (m, 1H), 5.04 (d, *J* = 6.5 Hz, 2H), 4.26 (t, *J* = 7.4 Hz, 2H), 1.99–1.81 (m, 2H), 1.34 (m, 14H), 0.90 (s, 3H); ^13^C NMR (100 MHz, MeOD) δ 138.0, 137.2, 136.9, 129.8, 128.0, 123.9, 123.7, 122.2, 58.3, 52.6, 51.0, 33.0, 31.1, 30.6, 30.5, 30.4, 30.1, 27.3, 23.7, 20.5, 18.4, 14.4. HRMS (HESI/Orbitrap) *m*/*z* calculated for C_22_H_33_ClN_2_ [CDIM]^+^: 325.2644, found 325.2638.

### 4.3. Growth Conditions and Strains

Bacterial strains were kept frozen in glycerol-lactose medium at −20 °C. Strains used were *Acinetobacter baumannii* (ATCC 19606), *Escherichia coli* (ATCC 25922), *Pseudomonas aeruginosa* (ATCC 27853), *Staphylococcus aureus* (ATCC 25923), *Staphylococcus epidermidis* (ATCC 14990), and *Streptococcus pyogenes* (ATCC 19615). Prior to the assay, they were seeded in BHI medium, incubated at 37 °C and spiked for 24 h for their metabolic activation.

### 4.4. Antibacterial Screening

The technique was performed by agar well diffusion (modified Kirby–Bauer) [41]. Briefly, 100 mm diameter plates were prepared with Mueller-Hinton agar (4 mm thick), mixed with 100 μL of a 0.5 McFarland standard of each bacterium under study (per plate), at an approximated temperature of 40 °C (prior solidification). Once the plates were solid, perforations of 8 mm diameter were made with a sterile material. Subsequently, 150 μL of the IL (solubilized in sterile water) were placed on each well at 0.125 mM, 0.25 mM, 0.5 mM, 1.0 mM, and 2.0 mM concentrations. A reference antibiotic was added as a positive control in the form of a Sensi-Disc to demonstrate the strain sensitivity (Mupirocin 5.0 μg, Clindamycin 2.0 μg, and Ceftazidime 30 μg), and sterile water was added as a negative control. Plates were incubated for 24 h at 37 °C in a bacteriological stove (DH4000 B II HILAB). Finally, the inhibition halo was read by a rule millimeter.

### 4.5. Determination of Minimum Inhibitory Concentration (MIC) and Half Maximum Inhibitory Concentration (IC_50_) by 96-Well Plate Microdilution

ILs antibacterial activity was assessed with a 96-well plate microdilution assay, a technique adapted from the methodology described by Eloff et al. [42]. The bacterial inoculum was obtained by incubating microorganisms in Mueller-Hinton broth for 24 h, adjusting it to a final concentration of 10^4^–10^5^ CFU / mL. ILs were solubilized in sterile water and the final concentrations for each IL was as follows 0.01562 mM, 0.03125 mM, 0.0625 mM, 0.125 mM, 0.25 mM, 0.5 mM, 1.0 mM and 2.0 mM. Each plate included bacterial growth (positive control) and culture medium (negative control) for 12 h at 37 °C. Microorganisms viability was determined by adding 20 μL of 3-[4,5-Dimethylthiazol-2-yl]-2,5Diphenyl-tetrazolium (MTT) dye at 0.5 mg/mL concentration for each well, and subsequently incubated for 3–4 h at 37 °C. Then, the formed crystals were solubilized with isopropanol buffer (10% Triton X-100 plus 0.1 N HCl in anhydrous isopropanol) obtaining the absorbance at 570 nm in a multi-plate reader. Results obtained from three independent experiments reported the lowest concentration of an IL, which prevents the microorganism’s visible growth (MIC), as well as the concentration inhibiting 50% of the growth (IC_50_).

### 4.6. Molecular Dynamics Simulations (MDs)

Molecular dynamics simulations (MDs) were performed to study the interaction mechanism between ILs and membranes. ILs [CMIM]Cl and [CDIM]Cl were selected for computational studies since they exhibit the worst and best biological activity, respectively. ILs were placed in a periodically repeating box containing explicit TIP3P [43], water molecules with a pre-equilibrated DOPC (1,2-Dioleoyl-sn-glycero-3-phosphocholine), and a lipid bilayer. Simulations were performed using the CHARMM force field [44] within the NAMD v2.12 software [45]. Force field parameters for [CMIM]Cl and [CDIM]Cl were assigned using the CGenFF force field [46] and the ParamChem website (https://www.paramchem.org/). The DOPC lipid bilayer, composed of 80 lipids (40 per monolayer), was hydrated on each side by 20 Å water slabs. Ligands were immersed in the TIP3P water by placing each ILs center of mass (com) at 30 Å from the lipid bilayer com. Systems were energy minimized (5000 steps), equilibrated for 1 ns, and simulated for 50 ns. Integration of the equations of motion was performed by using a NPT ensemble with a time step of 2 fs. Periodic boundary conditions were implemented in both systems. The SHAKE algorithm was applied to all hydrogen atoms; the van der Waals (VDW) cutoff was set to 9 Å. NoséeHoover Langevin piston was used to control the pressure at 1 atm. Overall, water, lipids, and ILs temperature were kept constant, coupling each group of molecules independently at 323 K, employing the NoséeHoover thermostat method with a relaxation time of 1 ps. Long-range electrostatic forces were considered by the particle-mesh Ewald (PME) approach. Data were collected every 1 ps during the MDs. VMD software [47] was used for molecular visualization and MD trajectories analysis.

### 4.7. ADME Properties Prediction

ILs ADME properties (absorption, distribution, metabolism, and excretion) were calculated using QikProp [48] to predict some physicochemical and pharmaceutical properties. Briefly, 44 descriptors were predicted for ILs, including molecular weight, van der Waals, surface areas of polar nitrogen, molecular volume, H-bond acceptors, H-bond donors, rotatable bonds, Log P (octanol/water), among others. From these descriptors, QikProp also assessed the compounds acceptability based on Lipinski’s rules [49].

## 5. Conclusions

A series of novel ILs derived from *N*-cinnamyl imidazole with different chain lengths under solvent-free conditions were synthesized. From the different compounds obtained were observed an increase in thermal stability as the alkyl chain length increased; the most effective against the Gram-positive bacteria studied was the IL longer alkyl chain ([CDIM]Cl) with a MIC value of 31.25 μM, nevertheless, this antibacterial activity was not evidenced against the Gram-negative bacteria studied. Molecular dynamics studies revealed a better interaction of the IL with the longer chain ([CDIM]Cl) with the lipid membrane surface, showing a passive diffusion mechanism towards the surface, which was not evidenced in the synthesized IL with the shorter chain [CMIM]Cl due to its lack of hydrophobicity. These findings along with the evidenced ADME properties, suggest that the novel IL 1-decyl-3-cinnamylimidazolium chloride are in the acceptable range established for human use and could be considered as a potent agent for the treatment of Gram-positive bacteria causative of SSTIs

## Figures and Tables

**Table 1:** Thermal decomposition temperatures (T_onset_) of the *N*-cinnamylalkylimidazolium ILs.

Ionic Liquids	Temperature (°C) Corresponding to		
	*T*_onset_/°C ^a^	*T*_10_/°C ^b^	*T*_50_/°C ^c^
[CMIM]Cl	61	118	245
[CHIM]Cl	76	148	251
[COIM]Cl	91	176	251
[CDIM]Cl	106	193	257

Decomposition temperatures (°C): ^a^ onset decomposition, ^b^ at 10% mass loss, ^c^ at 50% mass loss.

**Table 2:** Bacterial growth inhibition halos (mm) at different ILs concentrations in agar well diffusion assay.

	**Gram-Positive Bacteria**														
	***S. aureus***					***S. pyogenes***					***S. epidermidis***				
mM	0.125	0.25	0.5	1	2	0.125	0.25	0.5	1	2	0.125	0.25	0.5	1	2
Imidazole	−	−	−	−	−	−	−	−	−	−	−	−	−	−	−
[CMIM]Cl	−	−	+	++	++	−	+	++	++	++	−	−	+	++	++
[CHIM]Cl	−	−	++	++	++	−	++	++	++	++	−	−	++	++	++
[COIM]Cl	−	++	++	++	++	−	++	++	++	++	+	++	++	++	++
[CDIM]Cl	−	++	++	++	+++	−	++	++	++	+++	++	++	++	++	++
	**Gram-Negative Bacteria**														
	***E. coli***					***P. aeruginosa***					***A. baumannii***				
mM	0.125	0.25	0.5	1	2	0.125	0.25	0.5	1	2	0.125	0.25	0.5	1	2
Imidazole	−	−	−	−	−	−	−	−	-	−	−	−	−	−	−
[CMIM]Cl	−	−	−	−	++	−	−	−	-	−	−	−	−	−	+
[CHIM]Cl	−	−	++	++	++	−	−	−	−	−	−	−	−	−	+
[COIM]Cl	−	−	++	++	++	−	−	−	−	−	−	−	−	+	++
[CDIM]Cl	−	−	++	++	++	−	−	−	−	−	−	−	−	++	++

n = 3 (three independent experiments). (−), no inhibition; (+), < 15 mm; (++) 15–25 mm; (+++) > 25 mm.

**Table 3:** Minimum inhibitory concentration (MIC) and calculation of mean viability inhibition (IC_50_) of Gram-positive and Gram-negative bacteria.

	**MIC (μM)**			
	**[CMIM]Cl**	**[CHIM]Cl**	**[COIM]Cl**	**[CDIM]Cl**
*S. aureus*	250	125	62.5	31.25
*S. pyogenes*	125	62.5	62.5	31.25
*S. epidermidis*	250	125	62.5	31.25
*E. coli*	2000	500	500	250
*P. aeruginosa*	>2000	> 2000	> 2000	> 2000
*A. baumannii*	2000	1000	1000	1000
	**IC_50_ (μM)**			
	**[CMIM]Cl**	**[CHIM]Cl**	**[COIM]Cl**	**[CDIM]Cl**
*S. aureus*	137	106	32.9	27.3
*S. pyogenes*	41.6	38.1	25	19.4
*S. epidermidis*	149	55.7	38.5	31.2
*E. coli*	1380	340	290	210
*P. aeruginosa*	>2000	>2000	>2000	>2000
*A. baumannii*	1870	570	480	465

n = 3 (three independent experiments).

**Table 4:** Physicochemical descriptor calculated by QikProp simulation.

Ligands	MW (g/mol) ^a^	HB-D ^b^	HB-A ^c^	Rotable Bonds	Total SASA ^d^	MV (Å^3^) ^e^	PSA ^f^
[CMIM]Cl	200.283	0	4	3	491.174	799.137	5.203
[CHIM]Cl	270.417	0	4	8	587.938	1055.386	4.991
[COIM]Cl	298.470	0	4	10	607.754	1137.921	4.991
[CDIM]Cl	326.524	0	4	12	630.815	1217.397	4.991

^a^ Molecular Weight. 95% Drugs range (130.0–725.0); ^b^ Estimated number of H-Bonds that would be donated by the solute to water molecules in an aqueous solution. Values are averages that take over several conformations, so they can be non-integer. 95% Drugs range (0.0–6.0); ^c^ Estimated number of H-Bonds that would be accepted by the solute to water molecules in an aqueous solution. Values are averages that take over several conformations, so they can be non-integer. 95% Drugs range (2.0–20.0); ^d^ Total Solvent Accessible Surface Area (SASA). 95% Drugs range (300.0–1000.0); ^e^ Total solvent-accessible volume in cubic Å^3^ using a probe with 1.4 Å of radius. 95% Drugs range (500.0–2000.0); ^f^ Van de Waals surface area of polar nitrogen and oxygen atoms. 95% Drugs range (5.0–200.0). All predictions are based in a data set of 1712 drugs.

**Table 5:** Pharmacokinetics properties predicted by QikProp simulation.

Lead Ligands	QP log P (o/w) ^a^	QP log S ^b^	QP log HERG ^c^	QP log Kp ^d^	% HOA in GI ^e^	Qual HOA Model ^f^	Lipinski’s Rule of 5 Violations ^g^
[CMIM]Cl	1.859	−0.503	−6.903	−4.295	88	HIGH	0
[CHIM]Cl	3.511	−1.523	−6.728	−3.877	100	HIGH	0
[COIM]Cl	4.028	−1.573	−6.404	−3.748	100	HIGH	0
[CDIM]Cl	4.523	−1.684	−6.197	−3.628	100	HIGH	0

^a^ QP log P for octanol/water. 95% Drugs range (−2.0–6.5); ^b^ Predicted aqueous solubility. Log S, S in dm^3^ is the solute concentration in a saturated solution in equilibrium with the crystalline solid. (−6.5–0.5); ^c^ HERG K+ channel blockage; log IC_50_. Concern below −5.000; ^d^ QP log Kp for skin permeability. Kp in cm/h; ^e^ % model for Human Oral Absorption in Gastro Intestinal System. <20% in poor; ^f^ Quality of model for Human Oral Absorption in Gastro Intestinal System. >80% in high; ^g^ Maximum is 4.

## Supplementary Materials

The following are available online at http://www.mdpi.com/1420-3049/23/9/2354/s1, Figures S1–S5, S7, S9, S11 are ^1^H NMR spectrums (400 MHz, CDCl_3_) of compound **2**–**9**; Figures S6, S8, S10, S12 are ^13^C NMR spectrums (100 MHz, MeOD) of compound **6**–**9**; Figures S13–S16 are HRMS spectrum of compound **6**–**9**.
