# Supplementary material for: Novel Alkylimidazolium Ionic Liquids as an Antibacterial Alternative to Pathogens of the Skin and Soft Tissue Infections
Source: Molecules. 2018 Sep 14;23(9):2354. doi: 10.3390/molecules23092354 (PMC6225289; doi:10.3390/molecules23092354)
Supplement: Supplementary file 1 [file molecules-23-02354-s001.zip › molecules-335713-supplementary.pdf]

Supplementary Information

Novel Alkylimidazolium Ionic Liquids as an Antibacterial Alternative to Pathogens of the Skin and Soft Tissue Infections

Forero-Doria O.[1], Castro RI.[2,3], Gutiérrez M.[1], González-Valenzuela D.[4], Santos LS.[1], Ramírez D.[5], Guzmán L[6*].

[1] Instituto de Química de Recursos Naturales, Universidad de Talca, Talca, Chile.

[2] Multidisciplinary Agroindustry Research Laboratory, Universidad Autónoma de Chile, Talca, Chile.

[3] Carrera de Ingeniería en Construcción e Instituto de Ciencias Biomédicas, Universidad Autónoma de Chile, Talca, Chile

[4] Departamento de Salud Municipal de Hualañe, Maule, Chile

[5] Instituto de Ciencias Biomédicas, Universidad Autónoma de Chile, Talca, Chile

[6] Departamento de Bioquímica Clínica e Inmunohematología, Facultad de Ciencias de la Salud, Universidad de Talca, Talca, Chile.

* Corresponding author.

Departamento de Bioquímica Clínica e Inmunohematología

Facultad de Ciencias de la Salud

Universidad de Talca

P.O. Box 747, Talca, Chile

Tel.: +56-71-2200490

E-mail address: lguzman@utalca.cl

I. ^{1}H and ^{13}C NMR spectra

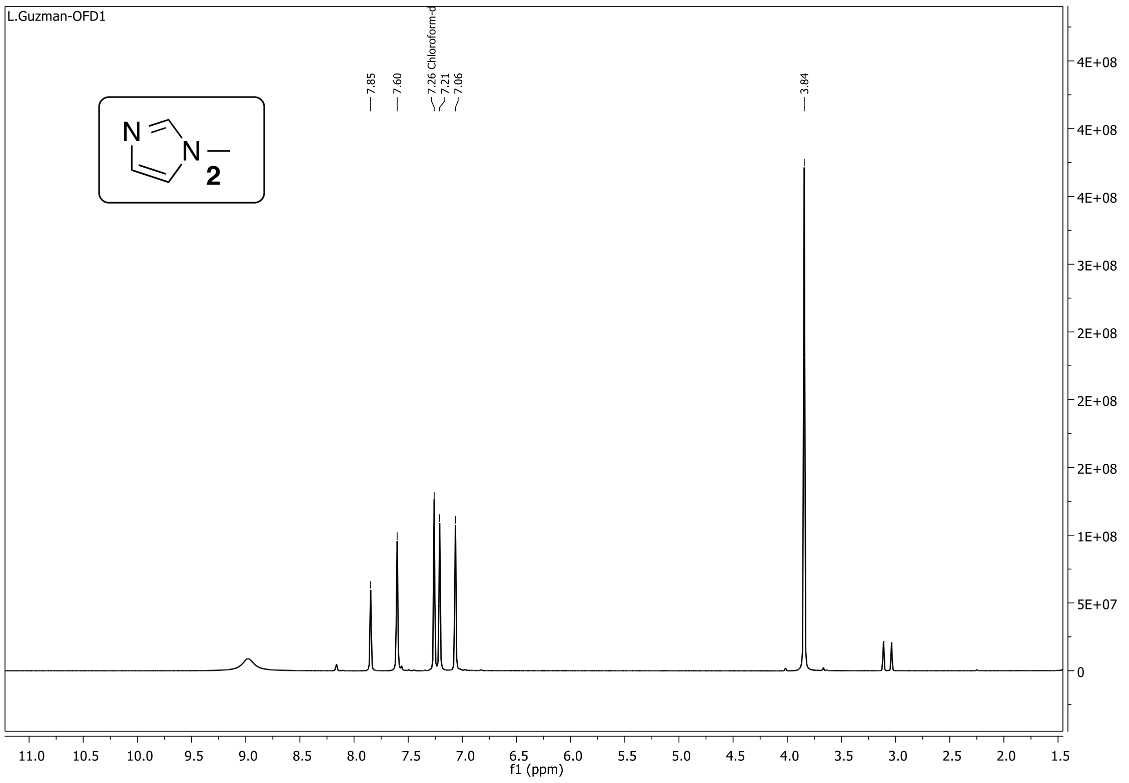

Figure S1. ^{1}H NMR spectrum (400 MHz, $CDCl_3$) of compound **2.**

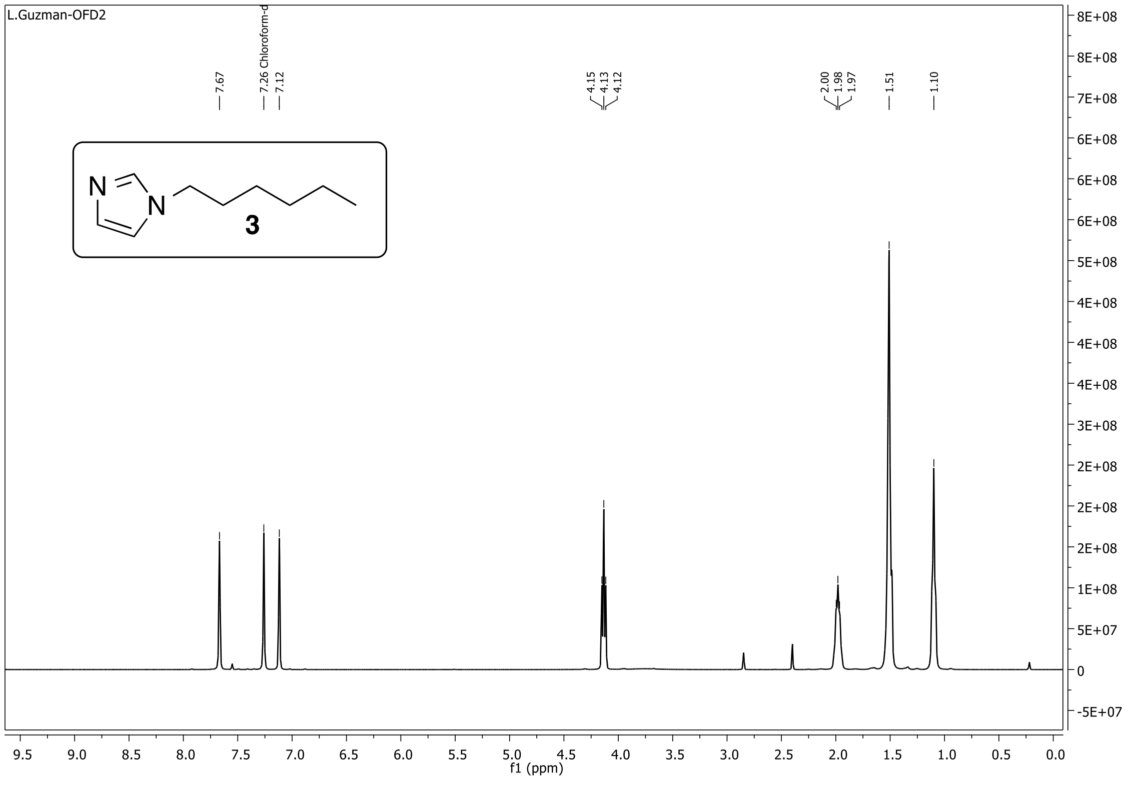

Figure S2. ^{1}H NMR spectrum (400 MHz, $CDCl_3$) of compound **3.**

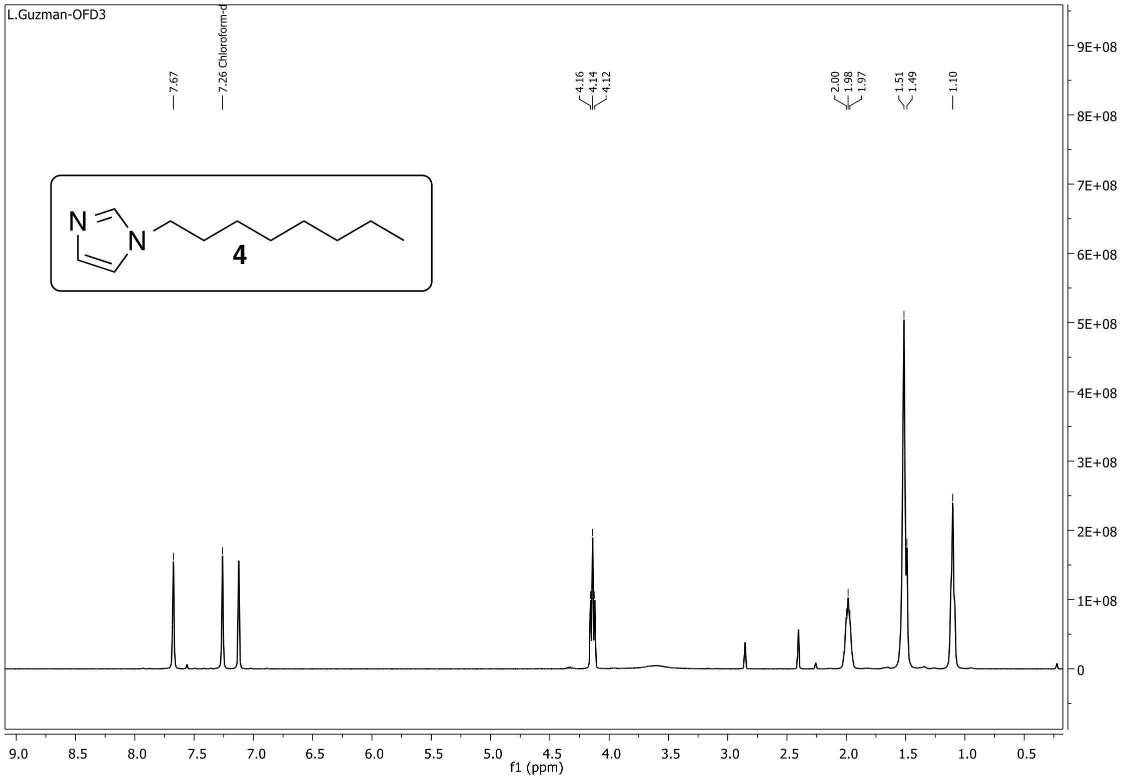

Figure S3. ^{1}H NMR spectrum (400 MHz, $CDCl_3$) of compound **4.**

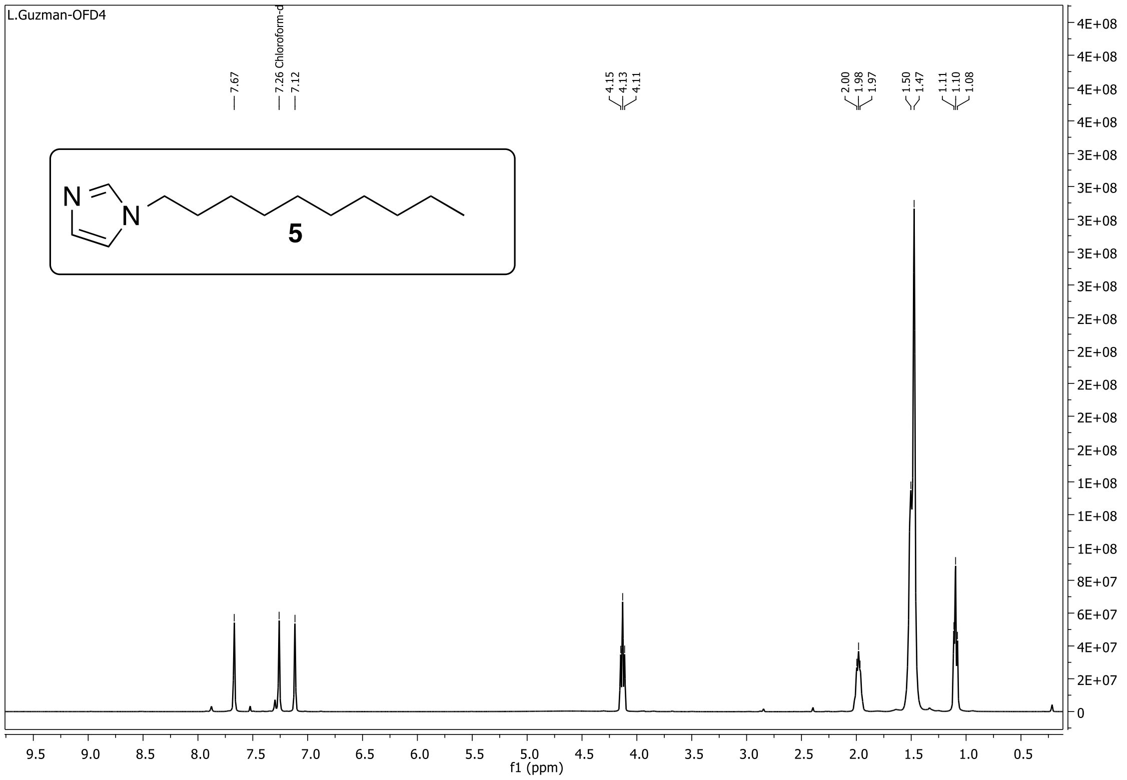

Figure S4. ^{1}H NMR spectrum (400 MHz, $CDCl_3$) of compound **5.**

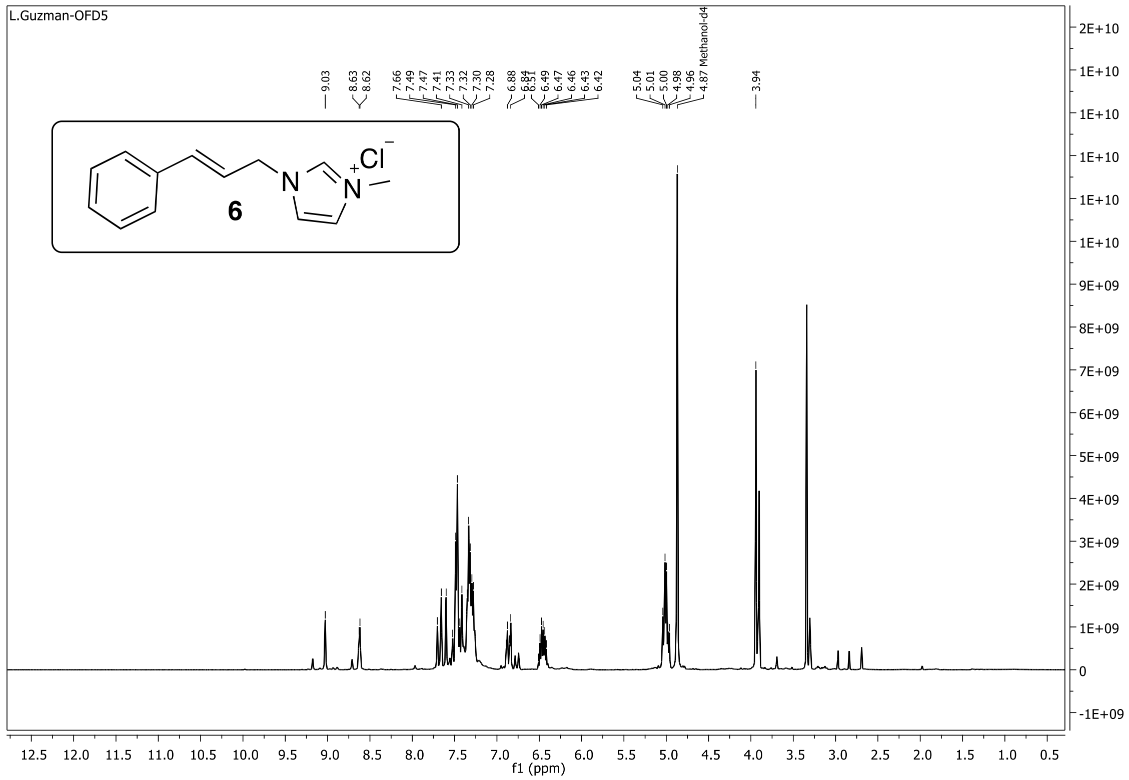

Figure S5. ^{1}H NMR spectrum (400 MHz, MeOD) of compound **6.**

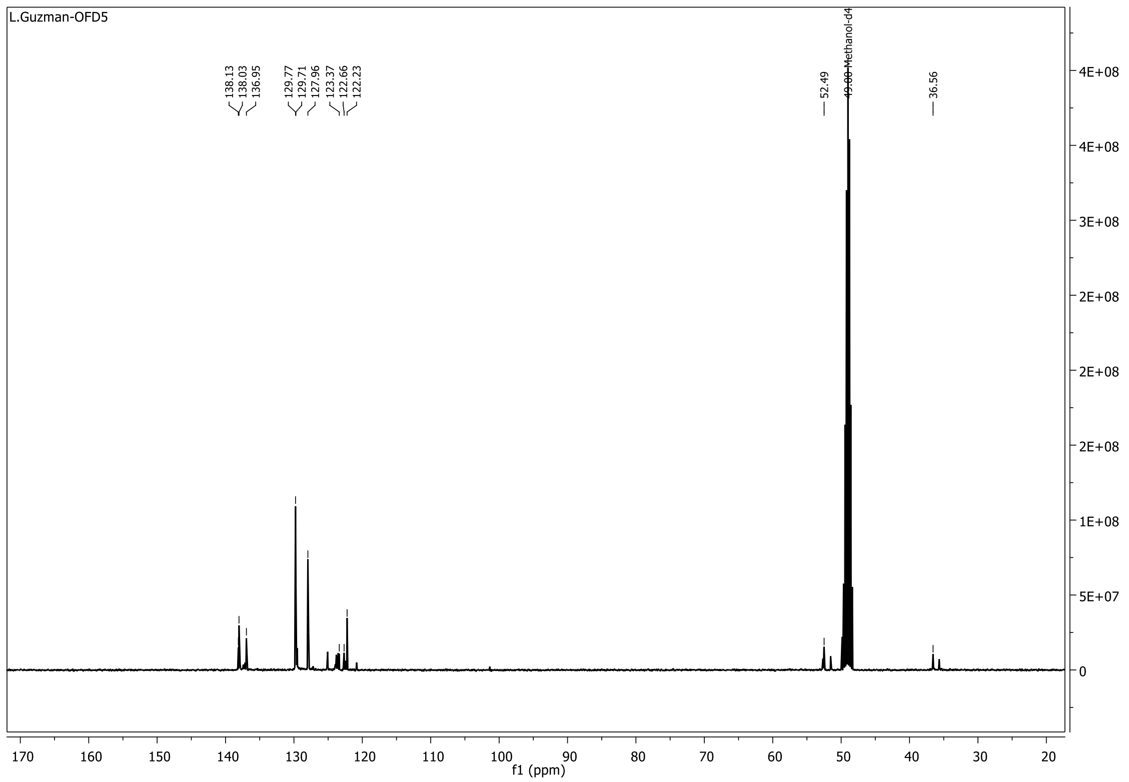

Figure S6. ^{13}C NMR spectrum (101 MHz, MeOD) of compound **6.**

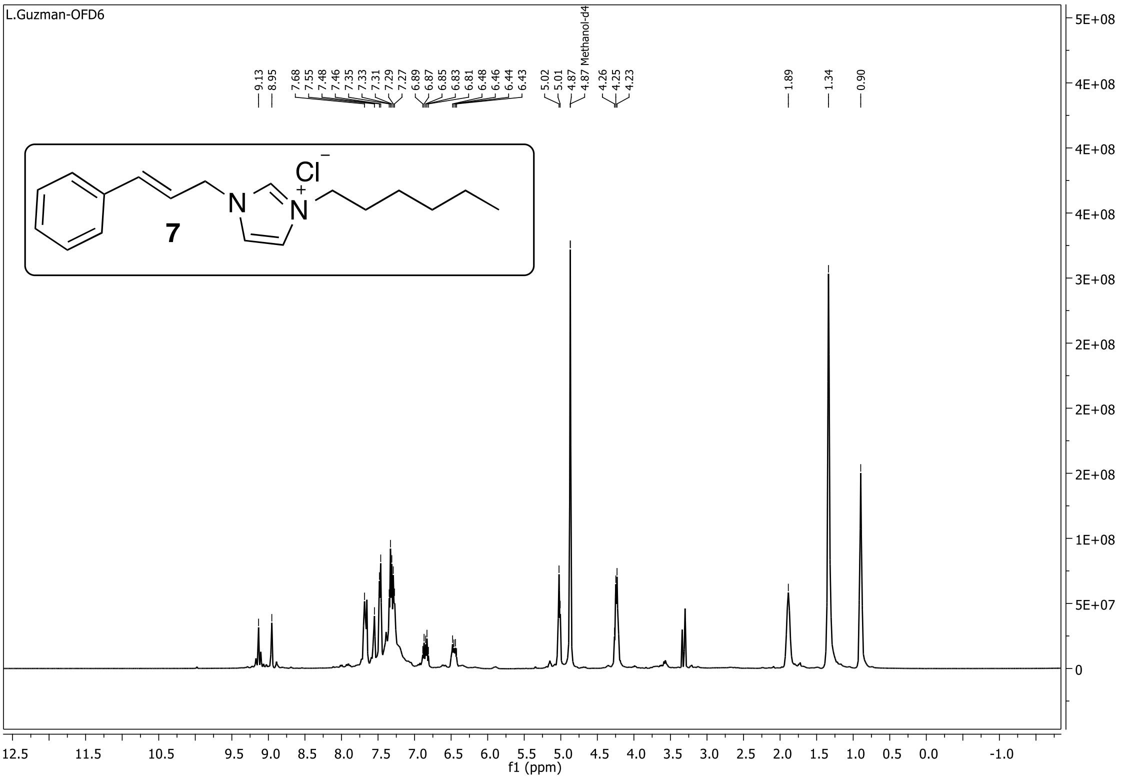

Figure S7. ^{1}H NMR spectrum (400 MHz, MeOD) of compound **7.**

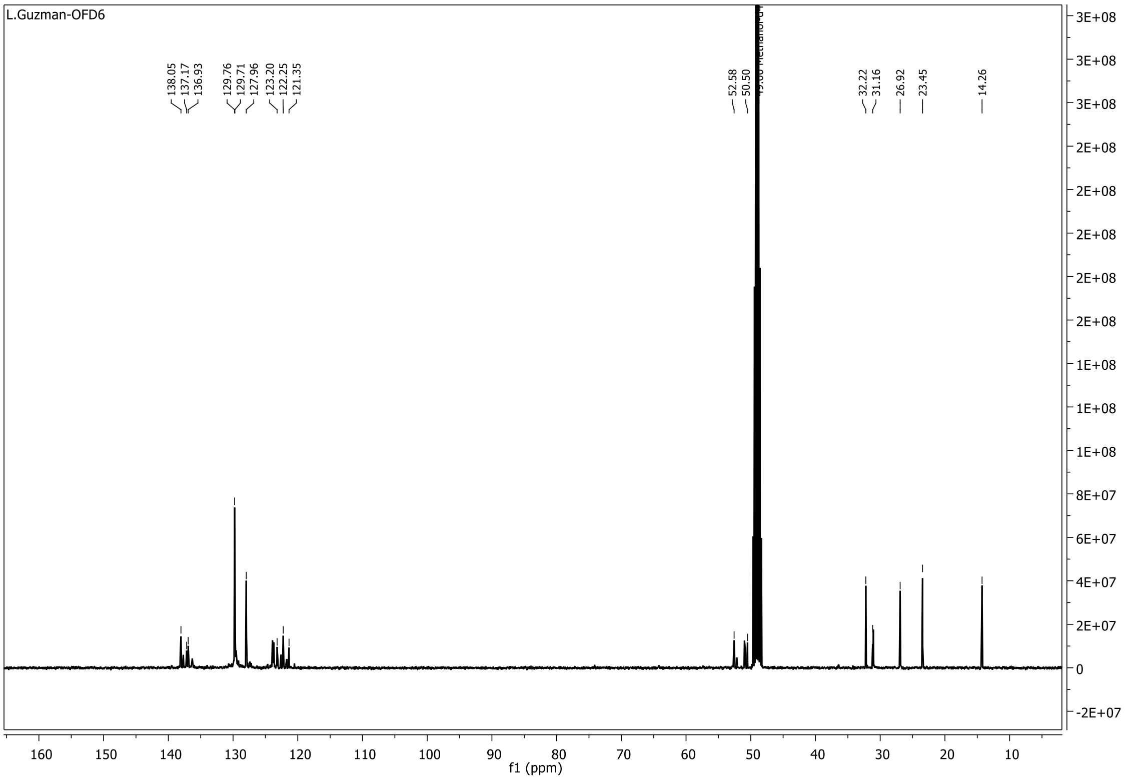

Figure S8. ^{13}C NMR spectrum (101 MHz, MeOD) of compound **7.**

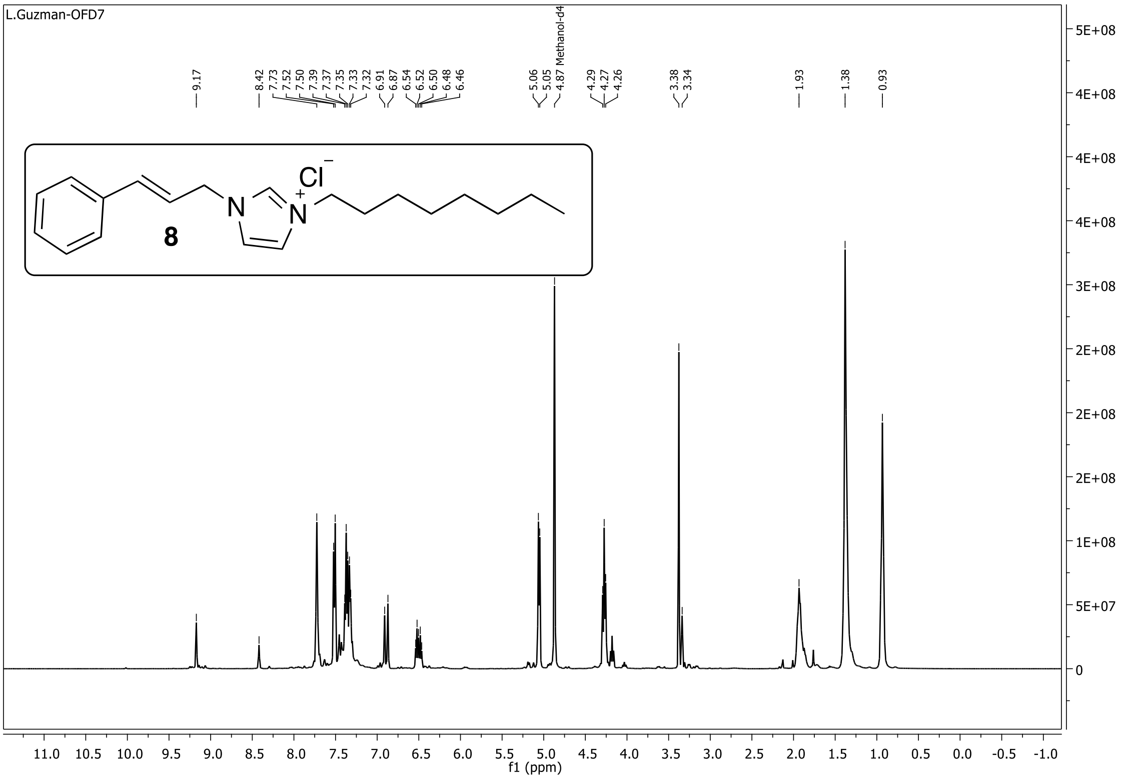

Figure S9. ^{1}H NMR spectrum (400 MHz, MeOD) of compound **8.**

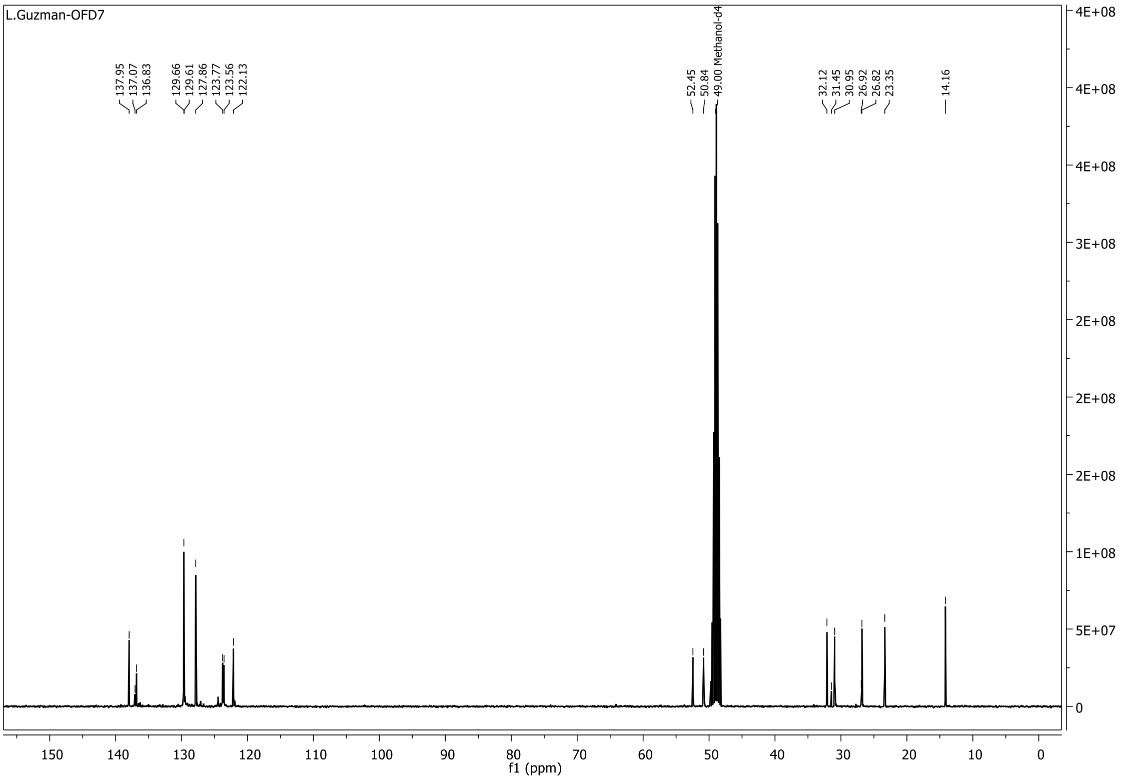

Figure S10. ^{13}C NMR spectrum (101 MHz, MeOD) of compound **8.**

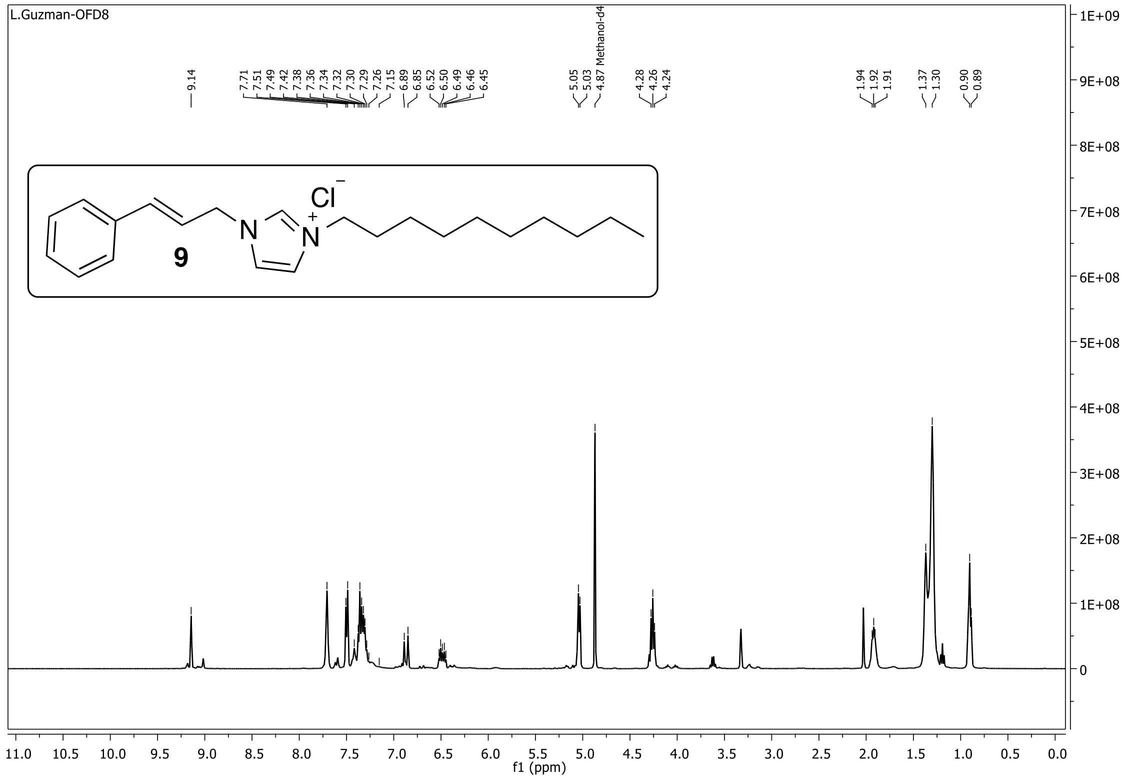

Figure S11. ^{1}H NMR spectrum (400 MHz, MeOD) of compound **9.**

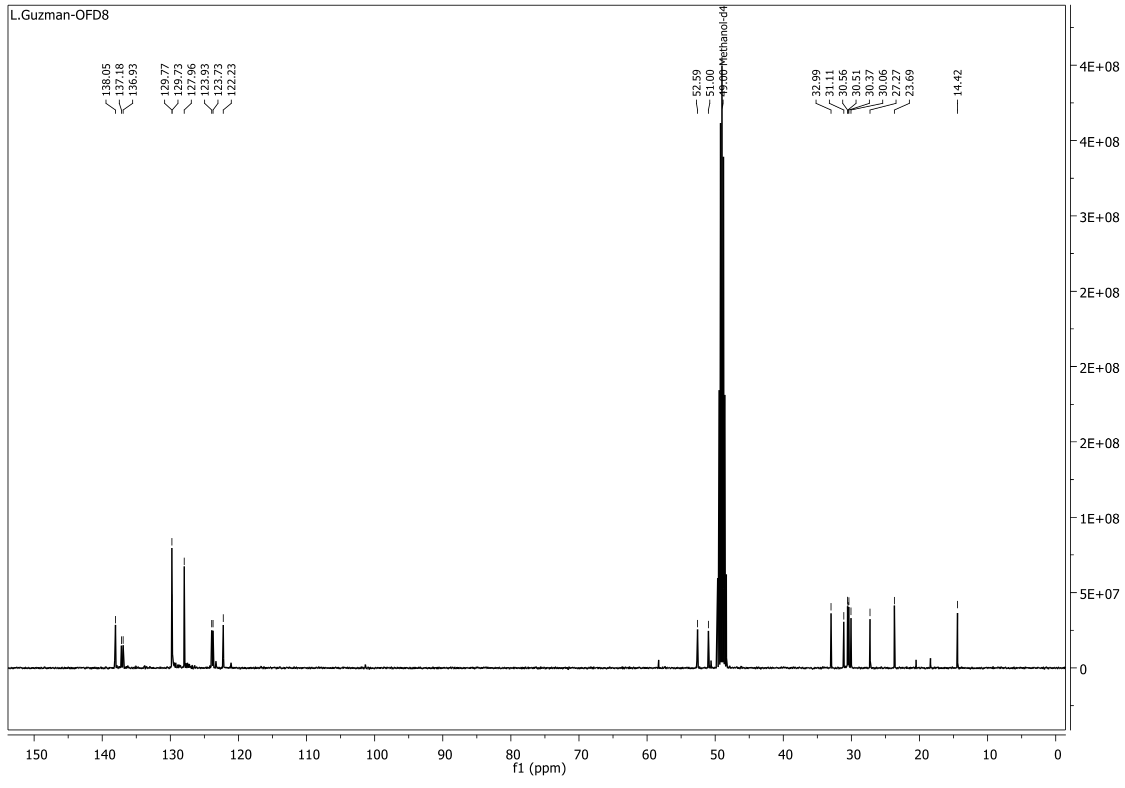

Figure S12. ^{13}C NMR spectrum (101 MHz, MeOD) of compound **9.**

II. HRMS spectra

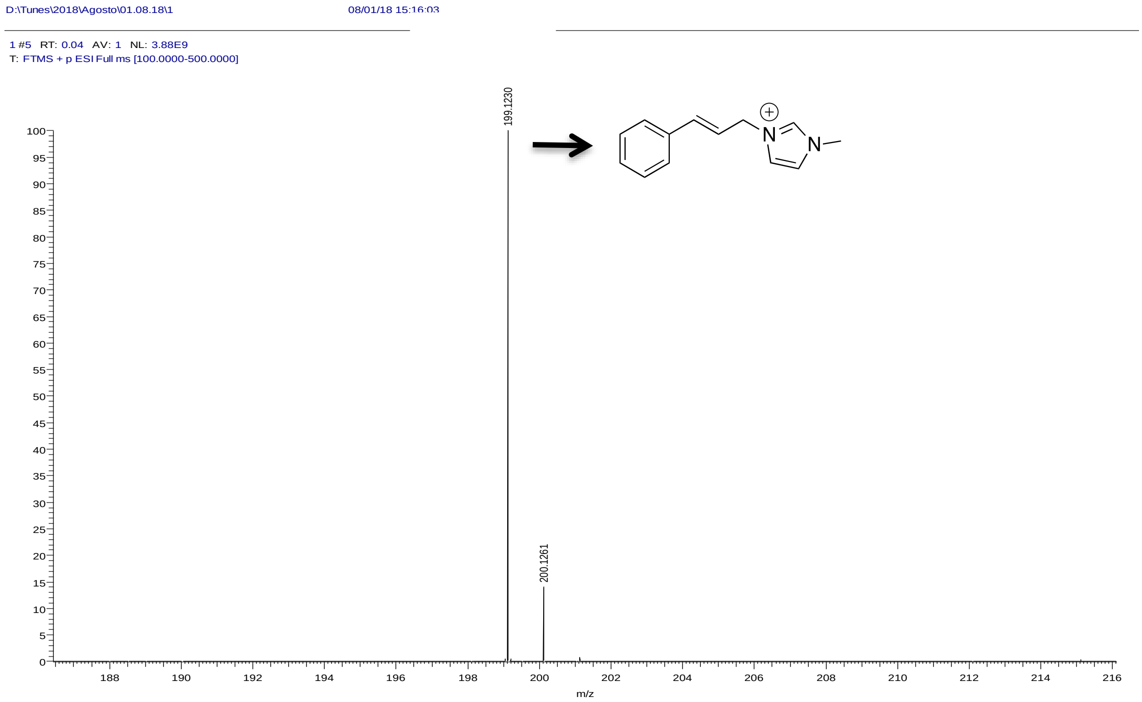

Figure S13. HRMS spectrum of compound **6**

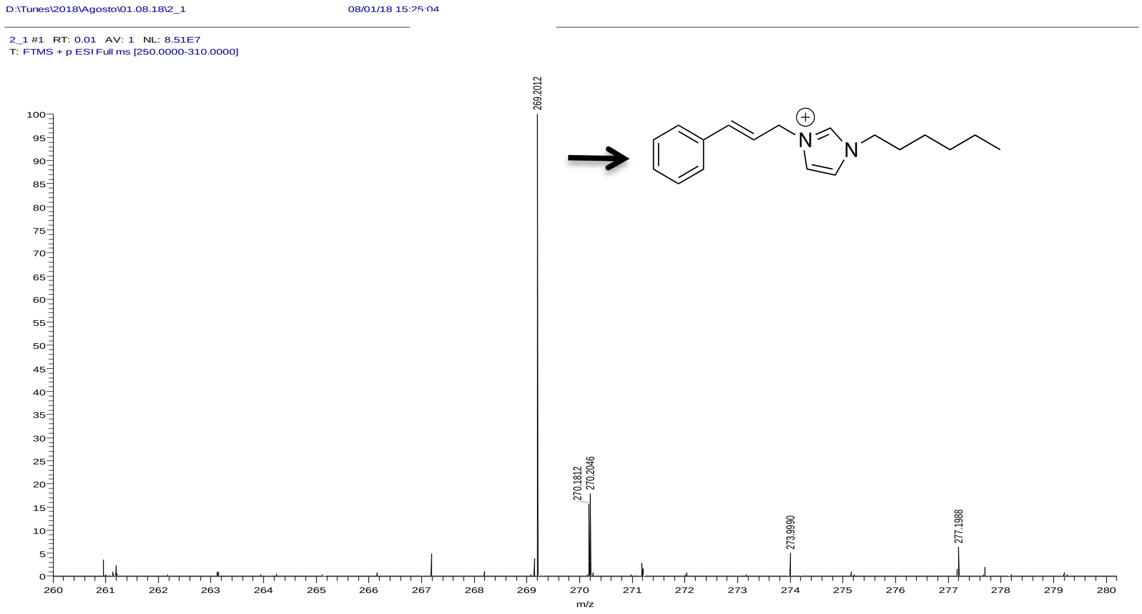

Figure S14. HRMS spectrum of compound **7**

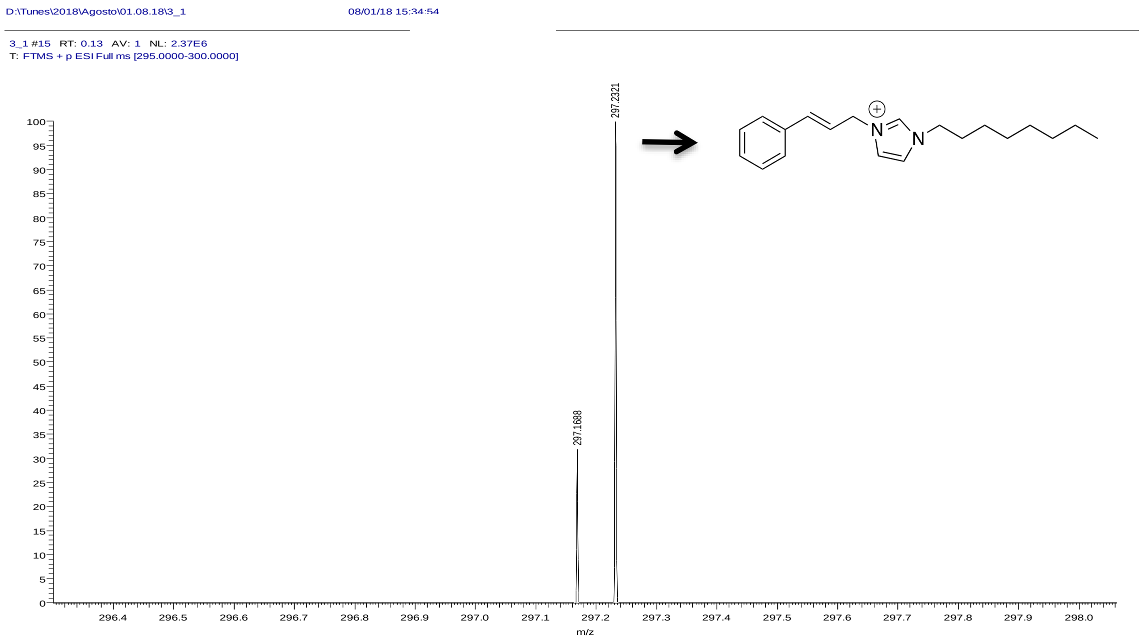

Figure S15. HRMS spectrum of compound **8**

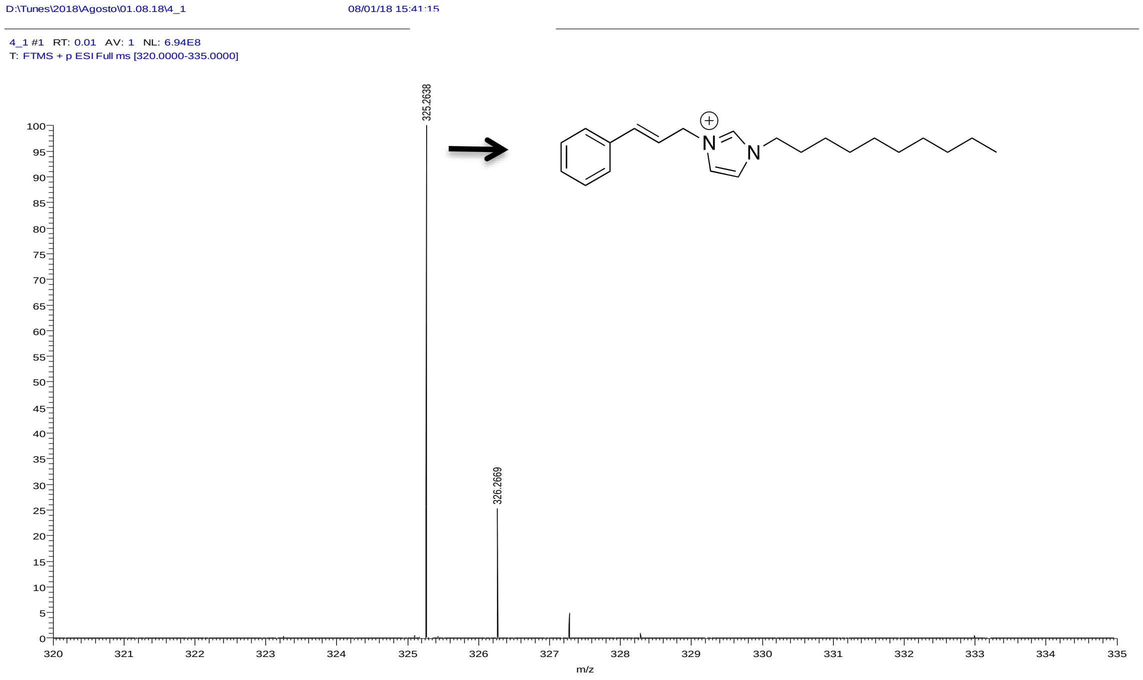

Figure S16. HRMS spectrum of compound **9**
